# Use of intraperitoneal fascial traction in robotic eTEP approach for complex ventral hernias: technique and outcomes

**DOI:** 10.3389/jaws.2026.16438

**Published:** 2026-08-26

**Authors:** Vivek Bindal, Dhananjay Pandey, Deepak Kumar, Shahiq Ahmed, Aakash Patel

**Affiliations:** Institute of Minimal Access, Bariatric and Robotic Surgery, Max Super Specialty Hospital, Delhi, Uttar Pradesh, India

**Keywords:** botulinum toxin A (BTA), fasciotens, intraoperative fascial traction (IFT), loss of domain (LoD), robotics

## Abstract

**Background:**

Tension-reduced midline closure remains a key challenge in the minimally invasive repair of large and complex ventral and incisional hernias. Commonly employed adjuncts, such as botulinum toxin A (BTA), progressive pneumoperitoneum (PPP), and component separation (CS), increase treatment complexity and may be associated with additional morbidity. Intraoperative fascial traction (IFT) has been described in open surgery to facilitate fascial medialisation; however, data related to its application in minimally invasive and robotic approaches supplementing IFT are scarce.

**Methods:**

A retrospective analysis was conducted of six patients who underwent robotic-assisted enhanced-view totally extraperitoneal (eTEP) ventral hernia repair with adjunctive IFT between June 2024 and March 2025. A comprehensive preoperative workup was performed, which included clinical evaluation and computed tomography (CT) imaging. The width of the fascial defect was meticulously measured intraoperatively before and after traction. For patients with transverse defects exceeding 15 cm, preoperative BTA injections were administered.

**Results:**

The mean pre-traction defect width was 15.8 ± 5.4 cm and reduced to 8.9 ± 3.2 cm following traction, achieving a mean medialisation of 6.9 ± 1.8 cm. Tension-reduced midline closure was achieved in all patients, without the need for CS or conversion to open surgery. Postoperative recovery was uneventful, with low pain scores, early ambulation, and no surgical site complications or 30-day readmissions.

**Conclusion:**

IFT is a feasible and effective adjunct in robotic eTEP repair of large ventral hernias. When combined with robotic precision, it facilitates closure without undue fascial tension while preserving abdominal wall integrity and avoiding CS. Larger-scale studies are warranted to validate these findings.

## Introduction

Minimally invasive surgery (MIS) has revolutionised the management of complex hernias, offering reduced morbidity, faster recovery, and superior cosmetic outcomes compared to open approaches [1]. Despite these advantages, achieving tension-free fascial closure in ventral hernias remains a significant challenge, particularly in cases involving large defects or loss of domain (LOD), where conventional closure techniques often lead to excessive tension, increased recurrence rates, and postoperative complications [2].

Several preoperative and intraoperative strategies have been developed to address these challenges. Techniques such as Progressive Preoperative Pneumoperitoneum (PPP), Botulinum toxin A (BTA) injections, and Component separation (CS) procedures have been widely adopted to facilitate fascial approximation [3]. However, these methods are associated with limitations, including the need for extended preoperative preparation, increased surgical complexity, and a higher risk of complications such as seroma formation, mesh infection, and abdominal wall dysfunction [4].

IFT has emerged as an innovative technique that allows for the controlled elongation of the myofascial structures, enabling primary closure without excessive tension [5]. This approach uses mechanical traction devices to gradually medialise the rectus complex, increase intra-abdominal volume, and facilitate a reduced-tension repair by lengthening the lateral muscle complex [6]. Unlike CS techniques, IFT preserves the structural integrity of the abdominal wall, in view of reducing the risk of denervation, functional impairment, and long-term hernia recurrence [5].

IFT has primarily been used in the open repair of large ventral hernias. The use of IFT in MIS (Robotic/Laparoscopic) can be very useful, but it is technically demanding. To date, only one case report has been published describing the use of IFT in Robotic Surgery. Bloemendaal et al. described the application of IFT in a robotic transabdominal retromuscular repair (r-TARUP) using an intra-abdominal approach. In contrast, our manuscript demonstrates the application of IFT in a robotic eTEP retrorectus approach with a distinct technical methodology and port strategy [7]. We report the first use of IFT in a robotic eTEP approach.

This article explores the application of IFT in MIS, detailing its surgical technique, clinical outcomes, and potential to redefine the paradigm of complex hernia repair.

## Materials and methods

A retrospective data collection was conducted at Max Super Speciality Hospital, Vaishali, between June 2024 and June 2025, enrolling six patients who had undergone surgical intervention for complex hernia repair. The preoperative assessment included taking a comprehensive medical history, performing a physical examination and carrying out standard laboratory investigations. A CT scan of the abdomen and pelvis was performed to aid in preoperative planning. Baseline variables including age, gender, body mass index (BMI), comorbidities, and other relevant hernia-related information were also collected. The Da Vinci Xi Surgical System (Intuitive Surgical, Sunnyvale, CA, USA) was utilised for robotic-assisted surgery. All surgeries were conducted by a single experienced surgeon.

All patients underwent an eTEP repair with IFT. In 5 out of 6 cases where the transverse defect exceeded 15 cm, preoperative administration of BTA was implemented to facilitate myofascial elongation. The size of the intraoperative defect was measured using an infant feeding tube before and after the application of fascial traction in order to evaluate its effectiveness in defect closure.

A total of 6 patients underwent IFT with Robotic Surgery. Five of these patients received preoperative BTA injections with an interval period of 4 weeks between the injections and the hernia repair. All patients received preoperative antibiotics and urinary catheterisation.

### Technique

The surgical procedure was performed using the Da Vinci Xi Surgical System in conjunction with the Fasciotens® Hernia System to facilitate IFT and subsequent fascial closure. This technique was applied to six patients presenting with large, recurrent incisional hernias. The surgical steps were standardised as follows:

Step 1 Patient Positioning and Robotic Setup.

**FIGURE 1:**
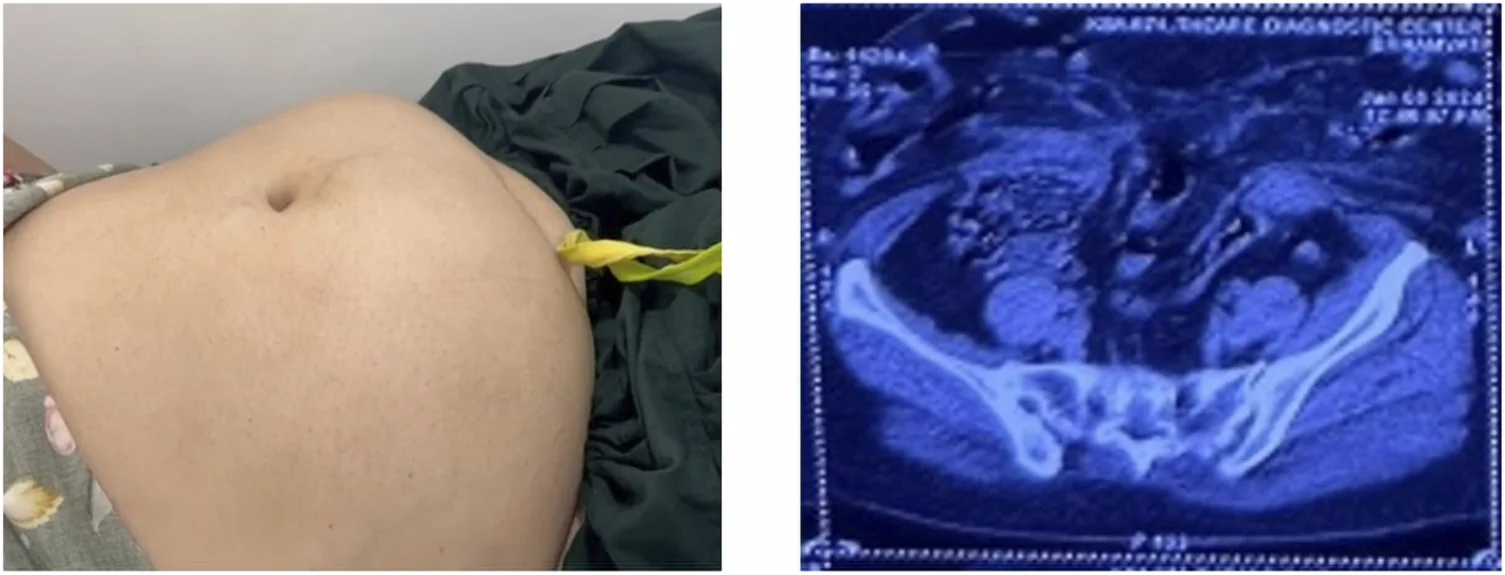
Shows clinical and radiological presentation of a complex ventral hernia. Axial CT scan (right) shows a 10.5 × 13.5 cm abdominal wall defect with loss of domain containing mesenteric fat and bowel loops, corresponding to the clinical mass seen in the patient photograph (left).

**FIGURE 2:**
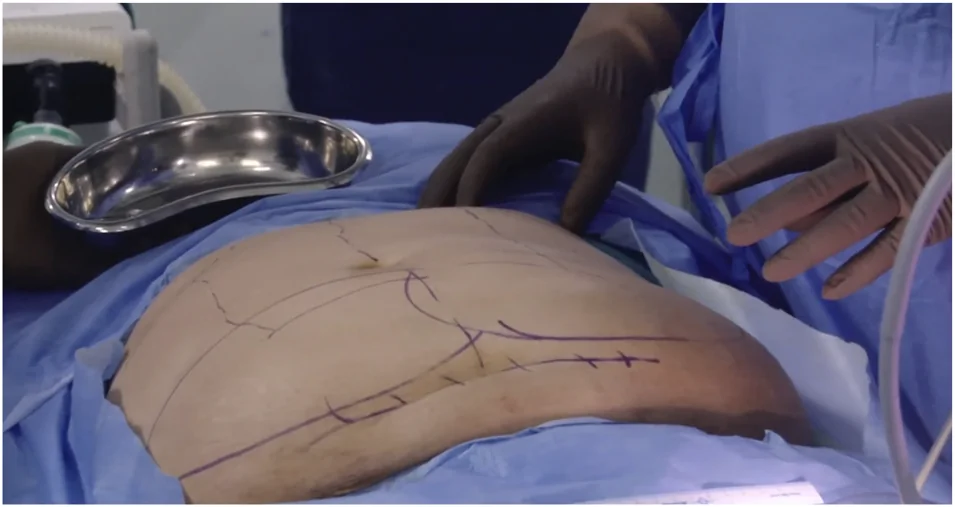
Intraoperative surface marking delineating the midline, subcostal margin, linea semilunaris, arcuate line, prior scar location, and projected extent of the hernial sac to guide surgical planning and fascial closure.

Under general anaesthesia, patients were placed in a supine position with their left arm tucked in to optimise abdominal wall exposure as shown in Figure 1 showing preoperative clinical and radiological assessment and Figure 2 showing intraoperative surface marking to guide surgical planning and fascial closure. The operating table was adjusted to ensure adequate reach for the robotic arms, to prevent instrument collision, and to allow application of the Fasciotens Hernia without hindering robotic arm movement.

Step 2 Creation of the retromuscular Space.

Access to the retromuscular space was achieved through a left upper abdominal incision, just medial to the assumed linea semilunaris, and a 5-mm optical trocar was utilised to gain entry into the potential plane to develop the working space. Insufflation was maintained at a target pressure of 14 mmHg to ensure optimal visualisation. Two additional 8-mm robotic trocars were placed under direct vision to facilitate instrument manoeuvrability, and a 5-mm optical port was then converted into an 8-mm robotic port.

Step 3 Rives-Stoppa repair.

The initial entry using an optical trocar into the left retrorectus space was made in the upper abdomen approximately 5–6 cm lateral to the midline, and the retrorectus space was expanded further using scope dissection. Two 8-mm secondary robotic trocars were placed just medial to the linea semilunaris at the level of the umbilicus and the left lumbar region to avoid any injury to the neurovascular bundles. The robotic patient cart was docked on the right side of the patient Crossover was performed into the opposite retrorectus space in the upper abdomen. Bilateral retrorectus dissection was performed further caudally. This process of division of the posterior rectus sheath (PRS) in the midline continued until the arcuate line, where the PRS becomes continuous with the fascia transversalis to enter the space of Retzius and Bogros [8].

Step 4 Hernia Sac Reduction and Adhesiolysis.

**FIGURE 3:**
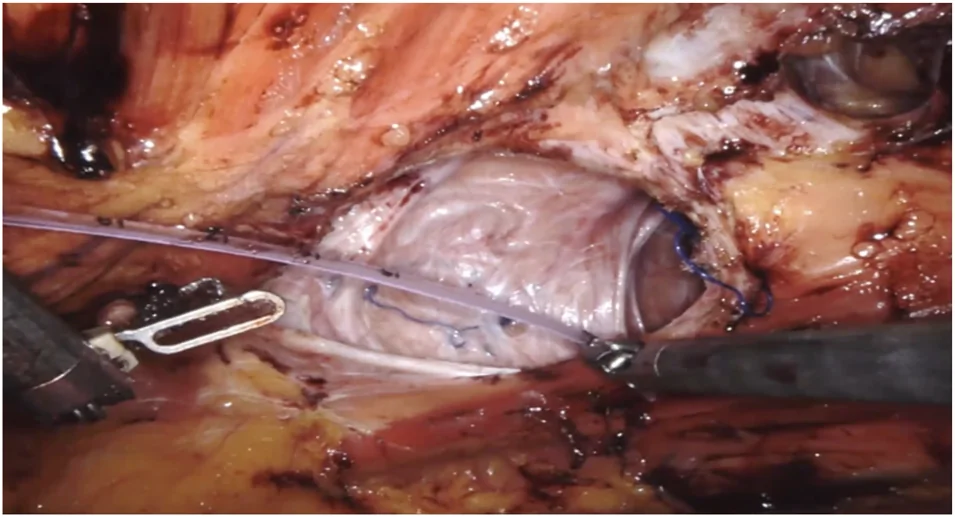
Shows intraoperative view demonstrating multiple anterior abdominal wall defects following creation of retro rectus (Rives Stoppa) plane. Defect dimensions and extent of the dissected space are assessed using an infant feeding tube for intraoperative measurement.

The hernia sac was meticulously dissected and reduced, followed by extensive adhesiolysis to free the fascial edges. Care was taken to preserve the integrity of the neurovascular bundles while ensuring complete mobilisation of the abdominal wall components as shown in Figure 3.

Step 5 Application of IFT.

**FIGURE 4:**
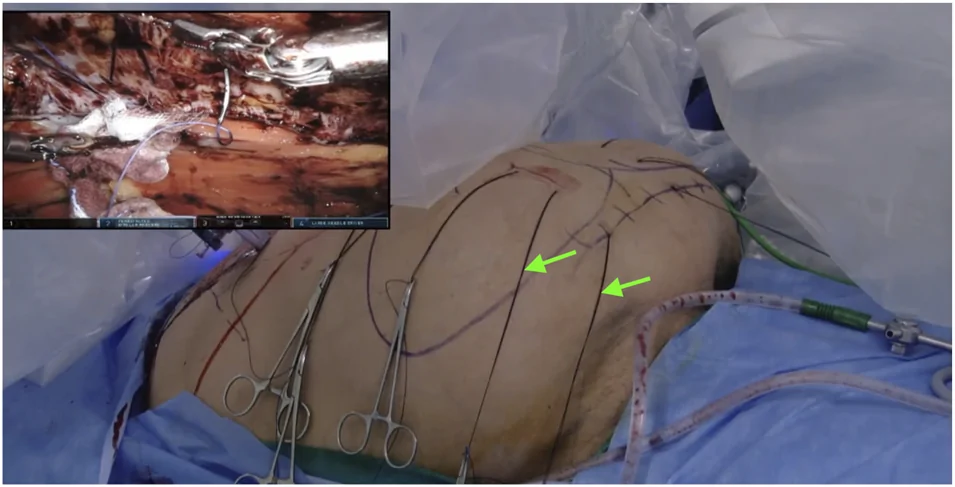
Showing intraoperative placement of traction sutures (green arrows) at fascial defect edges to facilitate controlled approximation (medialization). The inset demonstrates robotic needle driver assisted placement of suture loop at the fascial margin.

- The suture retention frame of the device was positioned above the patient’s abdomen and aligned with the fascial edges.
- The defect in the anterior rectus sheath was first measured in a cranio-caudal manner and divided into six equal segments.
- A Vicryl No. 1 suture was introduced in the working space from the robotic port and grasped by the robotic needle holder. It was then passed through the edge of the anterior rectus sheath at the first segment.
- The suture was looped into a 2-cm U-shaped segment, maintaining a 2-cm gap between each U-shaped loop and taking at least a 1-cm margin from the edge. Both ends of the suture loop were brought out of the working space transabdominally via a spinal needle suture passer.
- This process was repeated five more times to ensure the entire defect was evenly covered across all six segments. The procedure was then repeated on the contralateral side as shown in Figure 4.
- Traction was applied gradually over 30 min to allow for myofascial elongation.
- Anaesthesia ensured complete muscular relaxation during the traction period.

**FIGURE 5:**
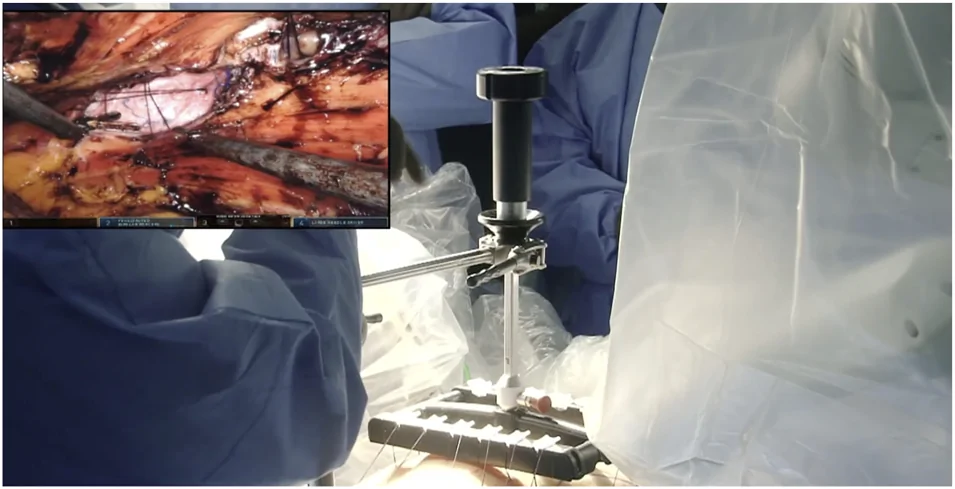
Fasciotens®Hernia system *in situ*, illustrating the mounted suture retention frame and adjustable traction controller used to modulate fascial tension intraoperatively.

The Fasciotens® Hernia system with calibrated vertical traction of 14 -18 kg was applied intraoperatively to generate progressive fascial tension, enabling medialisation of the rectus muscles as shown in Figure 5.

In one patient, we applied fascial traction to the PRS. Unfortunately, upon application of the fascial traction, the extraperitoneal space was obliterated, and the vision was compromised, which made this technique difficult to perform in order to repair the defect. In this scenario, we inserted another 5 mm port in the right upper abdomen, removed the fascial traction sutures under vision and then reapplied them to the anterior rectus sheath.

Importantly, this procedure was performed without the need to undock the robotic system, allowing for seamless intraoperative use of the device.

Step 6 Midline Closure and Mesh Reinforcement.

**FIGURE 6:**
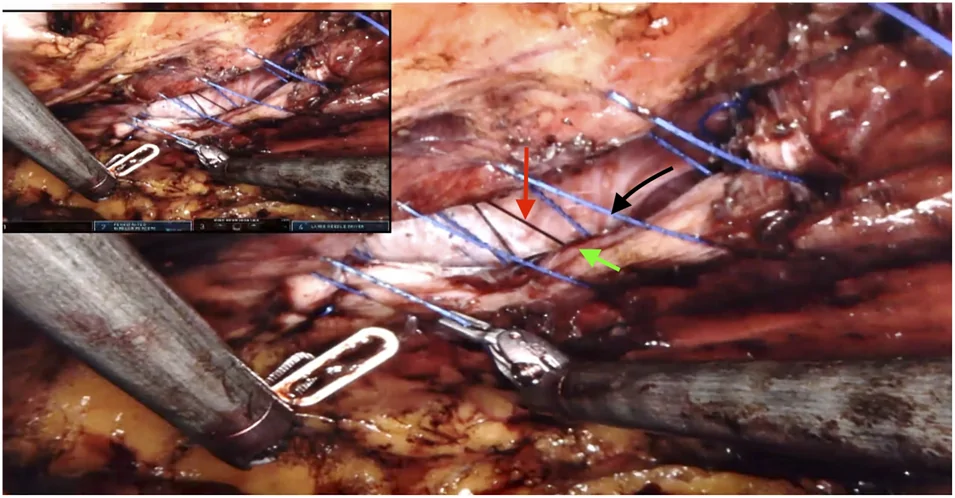
Intraoperative depiction of anterior rectus sheath approximation using absorbale barbed sutures for hernia defect repair. The black arrow identifies the intraabdominal barbed suture line, the red arrow indicates traction sutures anchored to the Fasciotens® suture retention system, and the green arrow delineates the medialized fascial margins of the defect.

Following sufficient medialisation, the peritoneum-posterior rectus sheath complex was approximated using running barbed delayed absorbable sutures (Stratafix 0 polydioxanone) to achieve a reduced-tension midline closure. A macroporous polypropylene mesh was placed in the sublay plane, ensuring adequate overlap beyond the defect margins. The mesh was secured with polypropylene 2-0 sutures wherever deemed necessary. Then, the Fascial edges of the Anterior Rectus Sheath were approximated using non-absorbable barbed sutures, and intra-abdominal pressure was indirectly assessed (without pneumoperitoneum) via airway pressure monitoring to ensure that the closure was tension-free as shown in Figure 6.

Step 7 Closure and Postoperative Management.

The robotic instruments were removed, and the port sites were closed in layers. Patients were followed up postoperatively for complications, with a focus on pain management, wound healing, and early ambulation. Follow-up was performed on postoperative day 1, after 1 week, after 1 month, and then again after 3 months.

## Results

Six patients underwent robotic-assisted eTEP hernia repair facilitated by IFT using the Fasciotens® Hernia System. The mean age of the cohort was 54.2 ± 9.1 years, with a male-to-female ratio of 2:1. The average BMI was 28.5 ± 3.8 kg/m^2^. The majority of patients (83.3%) had incisional hernias, and two patients (33.3%) presented with recurrent hernias. Table 1 summarizes the Demographic characteristics.

**TABLE 1:** Demographic characteristics.

Variable	Value
Age, mean ± SD (years)	54.2 ± 9.1
Sex (M/F)	4/2
BMI, mean ± SD (kg/m^2^)	28.5 ± 3.8
Comorbidities	HTN (3), DM (2), COPD (1)
Type of hernia	Incisional (5), primary (1)
Recurrent hernia	2 (33.3%)

In our prospective case series involving six patients who received robotic-assisted eTEP hernia repair with the adjunct use of Intra-abdominal Fascial Traction (IFT), the perioperative and early postoperative outcomes were highly favourable. All surgeries were completed without conversion to an open technique or the need for re-intubation, underscoring the safety of the procedure and the effectiveness of the anaesthetic management during surgery.

The mean width of the pre-traction fascial defect was 15.8 ± 5.4 cm, which was successfully reduced to 8.9 ± 3.2 cm after fascial traction, achieving an average fascial medialisation of 6.9 ± 1.8 cm. This enabled tension-free midline closure without any signs of elevated intra-abdominal pressure. Table 2 summarizes the Perioperative Parameters of the patients.

**TABLE 2:** Perioperative parameters.

Variable	Value
Hernia width, mean ± SD (cm)	15.8 ± 5.4
Hernia length, mean ± SD (cm)	10.2 ± 4.6
Defects >15 cm with BTA, n (%)	5 (83.3%)
IFT applied in all patients	Yes
Surgical approach	Robotic eTEP + Fasciotens® hernia system
Intraoperative fascial medialisation (cm)	Mean reduction: 6.9 ± 1.8
Operating time, mean ± SD (minutes)	165 ± 22
Postoperative pain score at 24 h (0–10)	3.6 ± 0.8
Conversion to open surgery	0
First bowel movement (mean days)	1.5 ± 0.5
Ambulation (postoperative day)	Day 1 for all patients
Return to professional work (by day 21)	5 (83.33%)
Surgical site complications	None
Re-intubation required	None
30-day readmissions	0

Pain levels progressively declined in the immediate postoperative period, with a mean NRS score of 5.6 ± 0.5 at 6 h, improving to 3.8 ± 0.7 at 24 h, and 2.2 ± 0.6 by postoperative day 3, highlighting the efficacy of minimally invasive access and enhanced recovery protocols.

All patients ambulated within 24 h, with a mean time to first bowel movement of 1.5 ± 0.5 SD days. The average hospital stay was 2.5 ± 0.5 SD days, and no patients required readmission within the 30-day follow-up period. A standardised postoperative analgesia protocol was followed for all patients, consisting of intravenous paracetamol as routine baseline analgesia, with additional tramadol or diclofenac administered on an as-needed basis depending on postoperative pain assessment and patient requirements.

Return to routine daily activities occurred at a mean duration of 7.0 ± 1.3 days, and five out of six patients (83.33%) resumed professional duties by postoperative day 21. No postoperative complications were recorded.

## Discussion

In the context of large and complex incisional hernias, the concept of LOD is of paramount importance in preoperative assessment and surgical planning. LOD describes a condition in which a substantial volume of abdominal viscera has chronically herniated and become trapped in the hernia sac, resulting in a disproportionately reduced intra-abdominal cavity capacity [9].

In this clinical setting, IFT has emerged as a transformative adjunct in the surgical management of large ventral and incisional hernias, particularly those complicated by LOD. IFT is a technique designed to gradually elongate the abdominal wall by applying a controlled, symmetrical stretching force across the fascial edges, thereby facilitating their medialisation and subsequent tension-reduced closure. This controlled fascial traction induces progressive viscoelastic elongation and medialisation of the abdominal wall musculofascial components, thereby increasing abdominal wall compliance and facilitating tension-reduced midline closure in large ventral hernias [6].

Clinical evidence supports the efficacy of IFT in improving outcomes in abdominal wall reconstruction. In a prospective study by Pous-Serrano et al., IFT—when combined with preoperative progressive pneumoperitoneum and botulinum toxin A—enabled effective closure in patients with significant LOD without the need for CST, with a low rate of major complications [10]. Similarly, Moreno-Egea and Carrillo-Alcaraz reported that the use of fascial traction techniques significantly improved fascial approximation, minimised the need for extensive dissection, avoided the requirement of posterior component separation/transversus abdominis release, and reduced postoperative complications such as seromas and wound infections, thereby accelerating patient recovery [11].

Robotic-assisted surgery offers significant benefits in the management of complex and large ventral hernias. The robotic platform facilitates superior ergonomics, magnified 3D visualisation, and tremor-free articulation, all of which significantly enhance the surgeon’s ability to dissect in avascular planes such as the retromuscular or pre-peritoneal planes [12]. Moreover, intracorporeal suturing—an essential step in reconstructing the posterior sheath or performing transversus abdominis release (TAR)—is significantly easier and more precise with robotic instruments [13].

Our findings align with the retrospective outcomes reported in our earlier work, in which we compared laparoscopic and robotic-assisted eTEP repairs in a high-volume Indian centre. This study demonstrated that the robotic group had significantly better outcomes across multiple parameters, including reduced operating time, lower postoperative pain scores at 24 h and 14 days, and improved wellbeing scores. Importantly, a lower need for CS was also observed in larger defects managed robotically, further supporting the role of robotic surgery in achieving functional closure without increased morbidity [14].

Combining robotic surgery with IFT provides exponential benefits: the precise tension control of IFT is complemented by the visual and technical advantages of robotics, allowing for real-time adjustments during medialisation and closure. The minimally invasive nature of robotic access also reduces overall tissue trauma, shortens recovery time, and minimises the need for component separation techniques [15].

The technical intricacies of implementing IFT within a robotic setup involve the intracorporeal placement of traction suture mechanisms along the linea alba or fascial edges, followed by their gradual tightening under direct visual control. While spatial limitations around the operating table—primarily due to the proximity of the robotic patient cart—pose a logistical challenge, we successfully executed IFT without undocking the robot.

To further optimise fascial compliance, the preoperative administration of BTA has been shown to paralyse the lateral abdominal wall musculature, leading to passive lengthening and decreased tension on the midline closure [16]. This technique, when combined with IFT, provides a biomechanically favourable environment for durable fascial closure.

A notable advantage of IFT is that it can obviate the need for component separation techniques (CST), which are traditionally used to approximate the rectus muscles, but which are associated with significant morbidity, including seroma formation, nerve injury, and wound complications [5]. By preserving the native integrity of the lateral abdominal wall, IFT offers a less invasive alternative that aligns with the principles of functional restoration and reduced surgical trauma.

By achieving primary midline closure through tension-reducing modalities such as IFT and BTA, the need for CST can be avoided, thereby improving both short- and long-term patient outcomes [17]. These results collectively underscore the procedural safety, enhanced recovery, and functional restoration achieved by using robotic-assisted repair in conjunction with the Fasciotens® Hernia System.

## Conclusion

The integration of IFT into minimally invasive and robotic-assisted hernia repair appears to be technically feasible and may serve as a useful adjunct for facilitating tension-reduced fascial closure in complex ventral hernias. Understanding the differential effects of fascial traction on anterior and posterior layers is essential for refining surgical techniques. Continued research and clinical experience will shape optimal management strategies for these challenging hernia cases.

## Data Availability

The raw data supporting the conclusions of this article will be made available by the authors, without undue reservation.
